# ASCT2 regulates glutamine uptake and cell growth in endometrial carcinoma

**DOI:** 10.1038/oncsis.2017.70

**Published:** 2017-07-31

**Authors:** A D Marshall, M van Geldermalsen, N J Otte, T Lum, M Vellozzi, A Thoeng, A Pang, R Nagarajah, B Zhang, Q Wang, L Anderson, J E J Rasko, J Holst

**Affiliations:** 1 Gene and Stem Cell Therapy Program, Centenary Institute, University of Sydney, Camperdown, New South Wales, Australia; 2 Sydney Medical School, University of Sydney, Sydney, New South Wales, Australia; 3 Origins of Cancer Program, Centenary Institute, University of Sydney, Camperdown, New South Wales, Australia; 4 Department of Tissue Pathology and Diagnostic Oncology, Royal Prince Alfred Hospital, Camperdown, New South Wales, Australia; 5 Cell and Molecular Therapies, Royal Prince Alfred Hospital, Sydney, New South Wales, Australia

## Abstract

Glutamine commonly becomes a conditionally essential amino acid in cancer. Glutamine is supplied to the cell by transporters such as ASCT2 (SLC1A5), which is frequently upregulated in multiple cancers. Here we investigated the expression of ASCT2 in endometrial carcinoma, and evaluated the contribution of ASCT2 to glutamine uptake and endometrial cancer cell growth. Analysis of human gene expression data showed that ASCT2 was significantly upregulated in both endometrioid and serous subtypes of endometrial carcinoma, compared to normal, age-matched endometrium. Furthermore, immunohistochemical staining of primary human endometrioid adenocarcinomas showed that tumours stain positive for ASCT2 in either a uniform or mosaic expression pattern, while normal adjacent glands appeared predominantly negative for ASCT2 staining. Chemical inhibition of glutamine transport by benzylserine or GPNA led to a significant decrease in endometrial cancer cell growth and spheroid cross-sectional area. ASCT2 knockdown recapitulated the decrease of cell growth and spheroid cross-sectional area in HEC1A cells, suggesting a reliance on ASCT2-mediated glutamine uptake. ASCT2 knockdown in Ishikawa cells led to lower glutamine uptake and cell growth, but did not affect spheroid area. Ishikawa cells express higher levels of the glutamine transporter SNAT1 compared to HEC1A cells, suggesting these cells may rely on both ASCT2 and SNAT1 for glutamine uptake. Since SNAT1 is also significantly upregulated in the endometrioid and serous subtypes, these data indicate that ASCT2 and SNAT1 could be used as markers of malignancy, and/or potential therapeutic targets in patients with endometrial carcinoma.

## Introduction

Endometrial carcinoma is a cancer arising from the lining of the uterus. There are various subtypes of endometrial carcinoma, with endometrioid adenocarcinoma representing about 80% of cases. Tumours of the endometrioid subtype are usually hormone responsive and generally have a good prognosis. The second most common subtype is serous carcinoma, which accounts for 5–10% of cases of endometrial carcinoma.^1^ The serous subtype is generally not hormone responsive, and is associated with a poorer prognosis. The treatment for endometrial carcinoma typically consists of surgery involving hysterectomy and salpingo-oophorectomy.^2^ Treatment of higher-grade tumours often also involves locoregional lymph node dissection as well as radiotherapy and/or chemotherapy.^3, 4^

In developed countries, endometrial carcinoma is the most common cancer of the female reproductive tract.^5^ Human papilloma virus (HPV)-associated cervical cancer is more prevalent in developing countries, due to the lack of effective screening programmes which have dramatically reduced rates in developed nations.^6^ However, this alone does not account for the high relative risk of endometrial carcinoma, which has an increased incidence of over 10-fold in first world countries.^7^ Several other cancers, including breast, ovary, prostate, colon, pancreas and kidney, have also shown a similar increase in incidence in ‘Western’ countries, irrespective of the patients’ genetic background. This increase has been associated with changes in diet, in particular an increase in dietary animal products which are inherently high in protein,^8, 9, 10^ suggesting that availability of nutrients such as amino acids may play a role in promoting cancer cell growth.

In mammalian cells, there are three classes of amino acids: non-essential, essential and conditionally essential. Non-essential amino acids can be synthesised by cells while essential amino acids must be obtained from external sources. Conditionally essential amino acids can be endogenously produced by cells, but under certain conditions, demand can outweigh supply. Cancer cells have a particularly high demand for amino acids due to their high proliferative rate, often upregulating amino acid transporters on their cell surface to facilitate increased cellular uptake (reviewed in Bhutia *et al.*^11^). Many cancer cells appear to be particularly dependent on one amino acid, glutamine. Cancer patients often develop glutamine depletion contributing to the development of cachexia, or disease-associated muscle wasting.^12^ The high demand for glutamine is likely due its contribution to numerous cellular pathways including cellular metabolism,^13^ in addition to its use in protein synthesis and activation of mTORC1.^14^ Glutamine can be metabolised to produce cellular energy via glutaminolysis. In this process glutamine is first converted to glutamate via glutaminase (GLS), which is subsequently converted into TCA cycle substrate α-ketoglutarate. This reaction can in turn activate mTORC1 signalling, further stimulating cell growth and survival.^15, 16, 17^ Glutamine can also be utilised as a nitrogen donor in pyrimidine and purine biosynthesis. In pyrimidine biosynthesis glutamine is metabolised via cytidine triphosphate synthetase (CTPS1/2) or carbamoyl-phosphate synthetase 2, aspartate transcarbamylase, and dihydroorotase (CAD). In purine biosynthesis glutamine is metabolised by phophoribosyl pyrophosphate amidotransferase (PPAT), guanosine monophosphate synthetase (GMPS) or phosphoribosylformylglycinamidine synthase (PFAS).^17, 18, 19^

Glutamine is actively transported across the cell membrane by amino acid transporters including ASCT2 (alanine-serine-cysteine transporter-2; SLC1A5), SNAT1 (sodium-coupled neutral amino acid transporter 1; SLC38A1) and SNAT2 (sodium-coupled neutral amino acid transporter-2; SLC38A2). We and others have shown that the small neutral amino acid transporter, ASCT2 is upregulated in many cancer types including: breast, prostate, skin (melanoma), colorectal, pancreatic, tongue and lung cancers.^20, 21, 22, 23, 24, 25, 26, 27, 28^ These data led us to hypothesise that ASCT2 may also be important for promoting and sustaining cell growth in endometrial cancer, where diet is known to be a contributing factor. Here we show that ASCT2 and SNAT1 are upregulated in both endometrioid and serous subtypes of endometrial carcinoma, compared to normal endometrium. We show that chemical inhibition of glutamine transport results in reduced cell growth and spheroid cross-sectional area *in vitro* in four separate endometrial cancer cell lines. Analysis of ASCT2 function by shRNA knockdown showed HEC1A cells rely on ASCT2-mediated glutamine uptake, while Ishikawa cells have only a partial reliance on ASCT2 function. These data demonstrate the importance of glutamine uptake in endometrial carcinoma, indicating that drugs targeting glutamine transporters such as ASCT2, and potentially SNAT1, may represent a novel therapeutic avenue.

## Results

### ASCT2 and SNAT1 are upregulated in endometrial carcinoma

**Figure 1:**
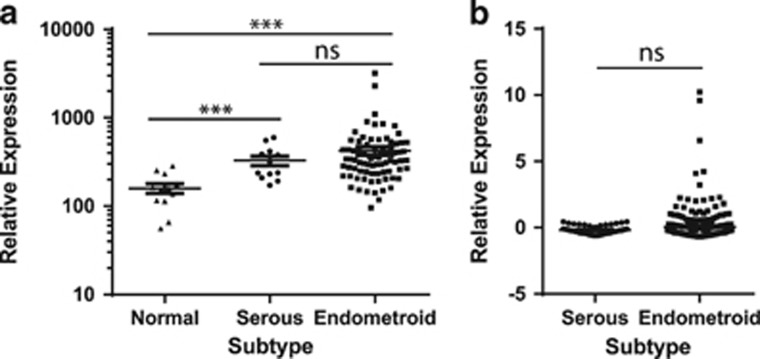
ASCT2 is upregulated in serous and endometrioid endometrial carcinoma subtypes. (**a**) ASCT2 expression in serous and endometrioid tumours compared to normal endometrium from age-matched controls derived from the Risinger *et al.*^29^ gene expression cohort. Normal *n*=12; Serous *n*=12, Stage I, Grade 3; Endometrioid *n*=79, Stage I, Grade 1–3. Mann–Whitney *U*-test: ***P<0.001, NS *P*>0.05. (**b**) ASCT2 expression in serous and endometrioid subtype tumours in the TCGA invasive endometrial carcinoma cohort.^30^ Serous *n*=52, Stage I–IV, Grade 3; endometrioid *n*=271, Stage I–IV, Grade 1–3; Mann–Whitney *U*-test: NS *P*>0.05.

To determine the expression levels of the amino acid transporter, ASCT2/SLC1A5, in endometrial carcinoma we compared gene expression in serous and endometrioid subtype tumours with normal age-matched endometrium using published gene expression data.^29^ These data showed a significant increase in ASCT2 mRNA expression in serous and endometrioid endometrial carcinoma samples in comparison to normal endometrium (*P*<0.001, Mann–Whitney *U*-test, Figure 1a). There was no statistically significant difference between ASCT2 expression in serous and endometrioid cell types. We also analysed the larger TCGA (The Cancer Genome Atlas) invasive endometrial carcinoma cohort^30^ and confirmed that there was no significant difference in ASCT2 expression between subtypes (Figure 1b). The expression of additional glutamine transporters, SNAT1/SLC38A1 and SNAT2/SLC38A2,^31^ was also investigated in these cohorts. SNAT1 was found to be significantly upregulated in endometrial carcinoma compared to normal endometrium (*P*<0.001, Mann–Whitney *U*-test, Supplementary Figure 1A). Moreover SNAT1 was significantly upregulated in serous carcinoma in comparison to endometrioid carcinoma (*P*<0.01 and *P*<0.001, Mann–Whitney *U*-test, Supplementary Figures 1A and B respectively). SNAT2 did not significantly vary between normal and malignant endometrium (Mann–Whitney *U*-test, Supplementary Figure 1C). However, there was a statistically significant increase in SNAT2 in serous carcinoma compared to endometrioid adenocarcinoma in the larger TCGA cohort (Supplementary Figure 1D).

**Figure 2:**
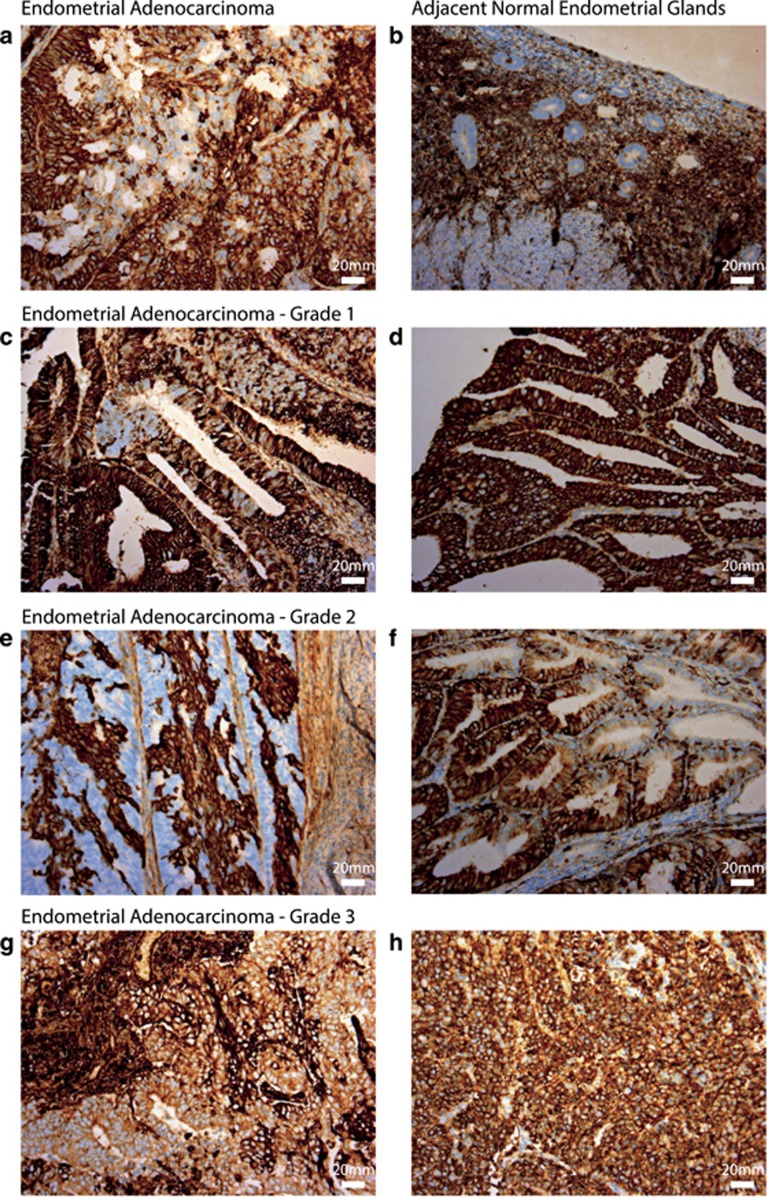
ASCT2 protein is upregulated in endometrial carcinoma compared to adjacent normal glands. Representative images demonstrating ASCT2 detected by immunohistochemistry in (**a**) endometrioid adenocarcinoma but usually not in (**b**) adjacent normal glands (**a**, **b** are representative case sample #005). Representative elevated tumour staining for ASCT2 is shown for (**c**, **d**) Grade 1 tumours (**c** is sample #003 and **d** is sample #016), (**e**, **f**) Grade 2 tumours (**e** is sample #002, **f** is sample #020), (**g**, **h**) Grade 3 tumours (**g** is sample #012 and **h** is sample #004). Staining was uniformly 3+ in 13/20 samples (representative images **d**, **h**) and variable between 0 and 3+ in 7/20 samples (representative images **a**, **c**, **e**, **f**, **h**). Scale bar represents 20 μm.

To investigate ASCT2 protein localisation and distribution in the endometrium and endometrial carcinomas, we performed immunohistochemistry in 20 primary human endometrial cancer samples (representative images are shown in Figures 2a–h). We observed that ASCT2 protein expression was high in the majority of tumours tested, where 13/20 showed 3+ staining (examples shown in Figures 2d and h). The remaining seven tumours had regions of variable staining across the tumour, with some negative areas and some areas of 3+ staining (examples shown in Figures 2a). In two cases there were regions of polarised staining, one in which apical cells were positive (Figure 2e) and one where basal cells showed increased staining. Where adjacent normal glands were present, 6/7 were negative for ASCT2 staining (Figures 2a and b), and in the remaining sample the majority of normal glands were negative, though some showed cells with ASCT2 positivity. In the same tumour there were some areas resembling complex atypical hyperplasia (CAH), which were also negative for ASCT2. A different sample showing some areas resembling CAH showed faint staining for ASCT2. Observations of all tumours tested are listed in Supplementary Table 1.

### ASCT2 is expressed in endometrial cancer cell lines

**Figure 3:**
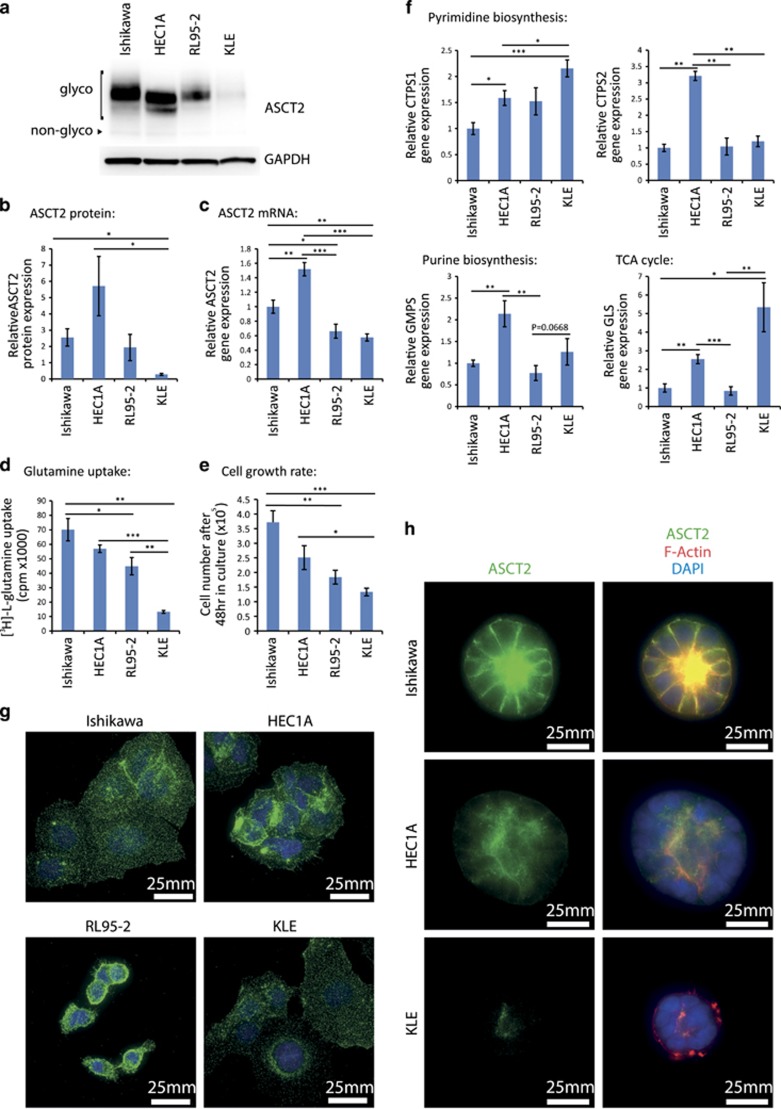
Expression of glutamine transporters and downstream enzymes in endometrial cancer cell lines. (**a**) Western blot showing relative ASCT2 expression in both non-glycosylated (non-glyco) and glycosylated (glyco) forms. GAPDH is provided as a loading control. (**b**) Densitometric analysis of western blots showing relative total ASCT2 expression in endometrial cancer cell lines. (**c**) RT–qPCR data showing the relative expression of ASCT2 mRNA, *n*=3. (**d**) [^3^H]-l-glutamine uptake was assessed in each cell line over 30 min. (**e**) Cell number of endometrial cancer cell lines 48 h after plating of 1 × 10^5^ cells, *n*=3. (**f**) Relative expression of pyrimidine biosynthesis enzymes CTPS1 and CTPS2, purine biosynthesis enzyme GMPS, and glutaminase GLS in endometrial cancer cell lines, *n*=5-6. Gene expression of other glutamine metabolism enzymes is shown in Supplementary Figure 2B. *P<0.05, **P<0.01 and ***P<0.001 Student’s *t*-test. (**g**) Immunofluorescence staining of Ishikawa, HEC1A, RL95-2 and KLE in 2D cell culture (green). Nuclei are counterstained with DAPI (blue). Scale bar represents 25 μm. (**h**) Immunofluorescence staining of Ishikawa, RL95-2 and KLE 3D spheroids after 8 days in culture with ASCT2 (green) rhodamine-phalloidin staining of F-Actin (Red) and DAPI staining of nuclei (blue). Scale bar represents 25 μm.

To determine if ASCT2 expression was present in endometrial cancer cell lines we performed Western blotting on four endometrial cancer cell lines: Ishikawa, HEC1A, RL95-2 and KLE. All showed expression of ASCT2, however the expression level and degree of ASCT2 glycosylation varied by cell line (Figure 3a). This protein expression corresponded with ASCT2 mRNA expression as determined by RT–qPCR (Figures 3b and c). To determine whether ASCT2 expression correlates with function, we assessed glutamine uptake in each cell line (Figure 3d). KLE cells, which had the lowest ASCT2 protein expression, showed the lowest glutamine uptake. However, despite higher ASCT2 expression, HEC1A cells took up similar levels of glutamine to Ishikawa and RL95-2 cells (Figure 3d). We therefore assessed expression of SNAT1 and SNAT2 glutamine transporters by RT–qPCR. Ishikawa and RL95-2 expressed higher SNAT1 and SNAT2 mRNA levels compared to HEC1A (Supplementary Figure 2A), suggesting SNATs may also be important for glutamine supply in these two cell lines. Overall glutamine uptake correlated loosely with cell growth assessed by cell counts (*R*^2^=0.7398), with Ishikawa cells, which express high levels of all three glutamine transporters, showing the highest rate of proliferation (Figure 3e). We also determined the expression of some downstream genes involved in the cellular metabolism of glutamine by RT–qPCR including the glutaminolysis enzyme GLS, pyrimidine biosynthesis enzymes: CTPS1, CTPS2 and CAD; and purine biosynthesis enzymes: PFAS, GMPS and PPAT. Of the rapidly proliferating cell lines, HEC1A had the highest level of expression of the majority of these enzymes, further suggesting a reliance on glutamine metabolism pathways (Figure 3f; Supplementary Figure 2B).

We next wanted to determine the subcellular localisation of ASCT2 in these cell lines and therefore performed immunofluorescence imaging. All cell lines showed cytoplasmic staining of ASCT2 with regions of cell surface localisation (Figure 3g). In order to better recapitulate the normal glandular structure of endometrial cancer cells *in vivo* we used 3D culture conditions^32^ to produce endometrial spheroids, and subsequently performed immunofluorescence. Under these growth conditions, ASCT2 predominantly localised to the apical cell membrane, though some faint basal localisation was also present in Ishikawa and HEC1A cells (Figure 3h).

### Chemical inhibition of glutamine transport inhibits endometrial cancer cell growth

**Figure 4:**
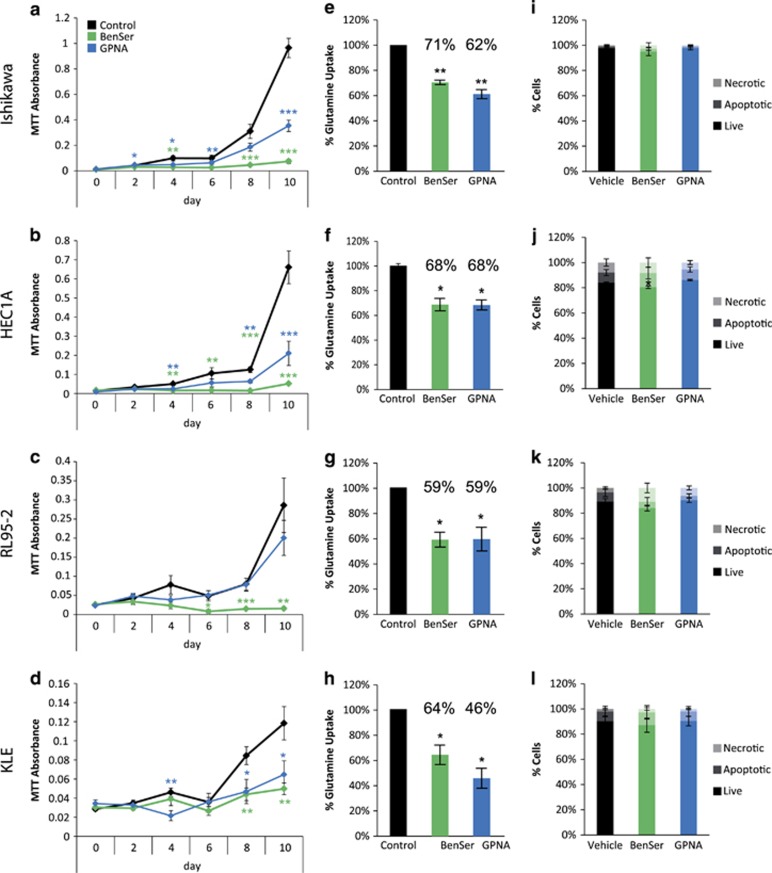
Chemical inhibition of ASCT2 reduces growth and glutamine uptake of endometrial cancer cell lines. MTT cell viability assays showing the effect of treatment with 10 mm BenSer or 1 mm GPNA in (**a**) Ishikawa, (**b**) HEC1A, (**c**) RL95-2, (**d**) KLE cell lines. [^3^H]-l-glutamine uptake following treatment with 10 mm BenSer or 1 mm GPNA for 30 min in (**e**) Ishikawa, (**f**) HEC1A, (**g**) RL95-2, (**h**) KLE cell lines. Annexin V/PI staining after 24 h of treatment with 10 mm BenSer or 1 mm GPNA in (**i**) Ishikawa, (**j**) HEC1A, (**k**) RL95-2, (**l**) KLE cell lines. Student’s *t*-test: *P<0.05, **P<0.01, ***P<0.001, NS *P*>0.05.

In order to determine if glutamine transport is critical for endometrial cancer cell growth we treated each endometrial cancer cell line with two chemical inhibitors: BenSer and GPNA. Treatment with BenSer was able to significantly reduce cell growth in all cell lines tested, while GPNA inhibited cell growth of Ishikawa, HEC1A and KLE cell lines but not RL95-2 (Figures 4a–d). BenSer was more effective than GPNA at inhibiting growth of Ishikawa (day 10 *P*=2.25 × 10^−5^), HEC1A (day 10, *P*=0.0247) and RL95-2 (day 10, *P*=0.0010), but inhibited growth similarly to GPNA in KLE cells. Both BenSer and GPNA similarly inhibited cellular [^3^H]-l-glutamine uptake to 71–59% or 68–46% of control depending on the cell line (Figures 4e–h). To determine if the changes in cell growth seen in MTT assays were due to changes in cell growth or survival, we performed Annexin V/Propidium Iodide flow cytometric analysis. There was no significant increase in cell death with drug treatment in any of the cell lines tested, except for a negligible but statistically significant decrease in the proportion of live cells in HEC1A (Figures 4i–l).

**Figure 5:**
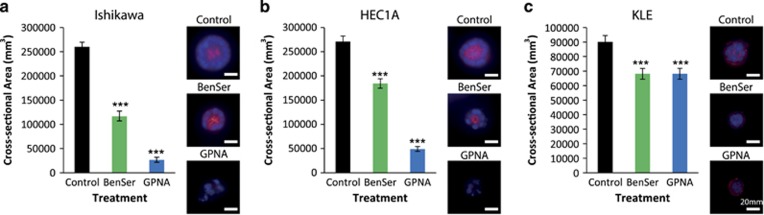
Chemical inhibition of ASCT2 reduces spheroid cross-sectional area. 3D spheroid cross-sectional area following treatment with 10 mm BenSer or 1 mm GPNA during 8 days of spheroid culture in (**a**) Ishikawa, (**b**) HEC1A and (**c**) KLE. Student’s t-test: ***P<0.001. Scale bar represents 20 μm.

We also tested whether BenSer and GPNA treatment altered spheroid growth. Both drugs were able to significantly reduce spheroid cross-sectional area in all three cell lines tested. In Ishikawa and HEC1A cells, GPNA was more effective than BenSer (*P*=5.43 × 10^−8^ and *P*=7.5 × 10^−8^ respectively) at reducing 3D cell growth, and also reduced the viability of spheroids, which often showed nuclear condensation by DAPI consistent with cell death (Figures 5a–c).

### ASCT2 is required for endometrial cancer cell growth

**Figure 6:**
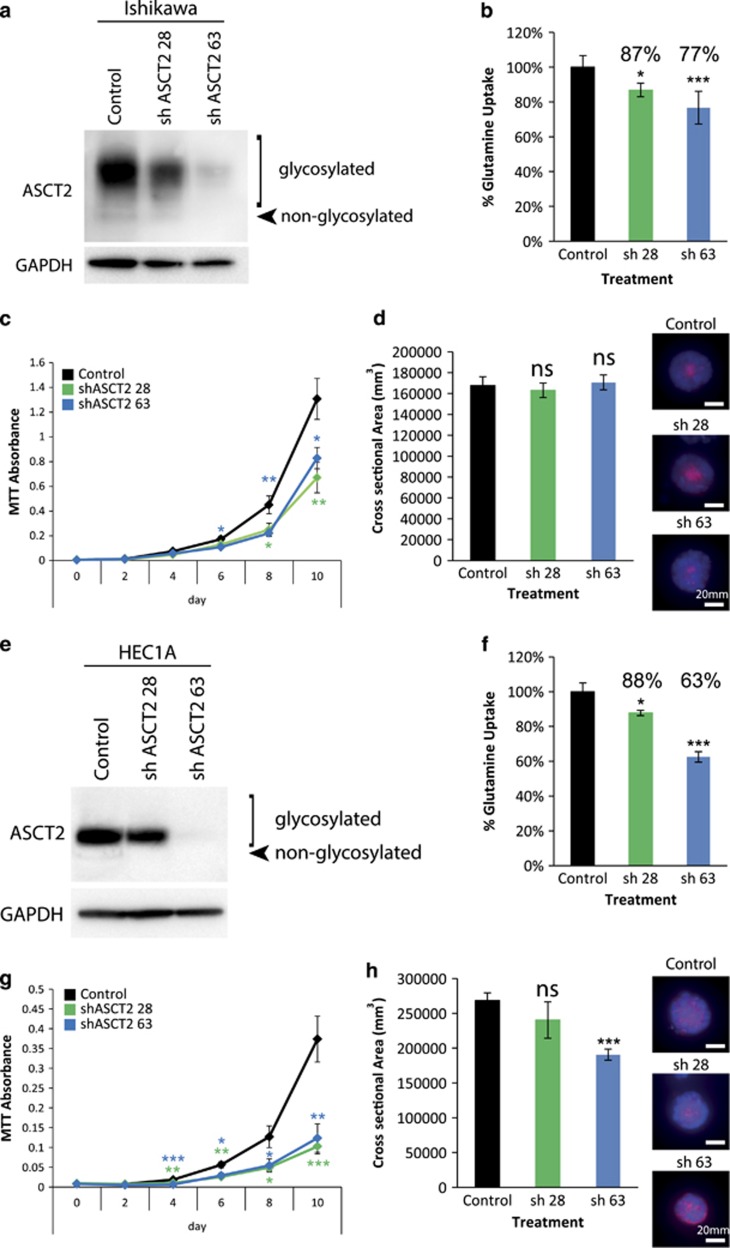
shRNA knockdown of ASCT2 largely recapitulates the results of chemical inhibition. (**a**) Western blot of shASCT2 28 (sh 28)- and 63 (sh 63)-mediated knockdown of ASCT2 in Ishikawa cells. (**b**) The effect of ASCT2 sh 28 and sh 63 on [^3^H]-l-glutamine uptake in Ishikawa cells. (**c**) MTT cell viability assays showing the effect of ASCT2 sh 28 and sh 63 on Ishikawa cells. (**d**) 3D spheroid cross-sectional area of Ishikawa cells expressing ASCT2 sh 28 and sh 63. (**e**) Western blot of shASCT2 28 and 63 mediated knockdown of ASCT2 in HEC1A cells. (**f**) The effect of ASCT2 sh 28 and sh 63 on [^3^H]-l-glutamine uptake in HEC1A cells. (**g**) MTT cell viability assays showing the effect of ASCT2 sh 28 and 63 on HEC1A cells. (**h**) 3D spheroid cross-sectional area of HEC1A cells expressing ASCT2 sh 28 and sh 63. Student’s t-test: *P<0.05, **P<0.01, ***P<0.001, NS *P*>0.05. Scale bar represents 20 μm.

Since BenSer and GPNA have been shown to affect multiple transporters including ASCT2, we next tested the specific role of ASCT2-mediated glutamine transport. ASCT2 knockdown was performed using two different shRNAs, shASCT2 28 (sh 28) and 63 (sh 63), in the two highest ASCT2-expressing endometrial cancer cell lines, Ishikawa and HEC1A. Both shRNAs produced a significant knockdown of ASCT2 expression in both cell lines, with sh 63 being the most effective shRNA by this measure (Figures 6a and e). Consistent with their ASCT2 expression levels, sh 28 reduced glutamine uptake by 13 and 12% and sh 63 reduced glutamine uptake by 23 and 37% in Ishikawa and HEC1A respectively (Figures 6b and f). Both sh 28 and 63 were able to significantly reduce cell growth in both Ishikawa and HEC1A, partially mirroring drug treatment results (Figures 6c and g). In addition, the more effective shRNA (sh 63) was able to reduce spheroid cross-sectional area in HEC1A but not in Ishikawa.

## Discussion

Endometrial cancer is considered a ‘Western’ disease occurring at increased rates in affluent developed countries. There are two main aspects of the ‘Western’ lifestyle that influence endometrial cancer development. The first is reduced childbearing (null or low parity), which is related to reproductive and hormonal factors. To exemplify this, early onset of menarche, late onset of menopause, infertility, irregular menstruation, unopposed hormone replacement therapy and tamoxifen use are also risk factors for endometrial cancer development.^7, 33, 34^ The second contributing factor is diet. Endometrial cancer has been associated with consumption of a diet high in animal protein.^9, 35^ Moreover, both obesity and diabetes are additional risk factors for endometrial cancer development.^36, 37, 38^ This suggests that nutrient availability may play a key role in the promotion of endometrial cancer development and/or progression.

Previously we have shown that the l-type amino acid transporter LAT1 plays a growth-promoting role in endometrial cancer.^39^ We showed that upregulated LAT1 is important for the uptake of the essential amino acid leucine, which in turn activates the mTORC1 pathway to promote the growth of endometrial cancer cells. LAT1 is a reciprocal transporter which imports essential amino acids, such as leucine, in exchange for efflux of other normally abundant amino acids, such a glutamine.^40^ However, cancer cells have high requirements for nutrients, making glutamine a conditionally essential amino acid in many cancers.^11^ This means that the levels of intracellular glutamine regulate the ability of LAT1 to promote growth of endometrial cancer cells. Glutamine uptake in cells is mediated by transmembrane proteins such as the amino acid transporters ASCT2, SNAT1 and SNAT2. ASCT2 upregulation has been implicated in several cancer types.^20, 21, 22, 23, 24, 25, 26, 27, 28^ In this study we have shown that ASCT2 and SNAT1 are upregulated in both the serous and endometrioid subtypes of endometrial carcinoma, compared to normal endometrium. Furthermore, we have shown the importance of glutamine uptake in four endometrial cancer cell lines, and that ASCT2-mediated glutamine transport contributes to cell growth in Ishikawa cells, and is essential for cell growth and 3D spheroid growth and survival in HEC1A cells.

Immunohistochemistry of primary human endometrial cancer cells showed that ASCT2 was highly expressed in endometrial cancer, in either a ubiquitous or mosaic staining pattern, in all 20 of the endometrioid adenocarcinoma cases examined. This is in stark contrast to adjacent normal glands which were negative for ASCT2 staining in 6/7 cases, with the remaining case showing staining in some isolated glands. Because these ‘normal’ glands are found near to malignant ones, the isolated ASCT2-positive ‘normal’ glands could contain precancerous molecular alterations that have induced ASCT2 gene expression. Collectively, these data indicate for the first time that ASCT2 immunohistochemical staining may be a useful pathological marker for endometrial malignancy. While we did observe an increase of ASCT2 expression in both serous and endometrioid subtypes of endometrial cancer, we did not observe a correlation between ASCT2 mRNA expression level and clinical outcome in analysis of the TCGA cohort. However, it remains to be seen whether the diversity of ASCT2 immunohistochemical staining has prognostic significance in endometrial carcinoma. In order to investigate this possibility further, a larger cohort including normal active and inactive endometrium, CAH and different subtypes of endometrial carcinoma, and associated clinical data would be required. This warrants investigation as ASCT2 detection by immunohistochemistry has prognostic significance in several cancer types.^23, 25, 26^ Hassanein *et al.*^41^ have performed preclinical studies using 4-[^18^F]-Fluoroglutamine PET as a non-invasive measure of ASCT2 expression in lung cancer. Such technology could also be useful in the diagnosis and staging of other ASCT2-positive cancers such as endometrial cancer.

Little is known about the role of ASCT2 in the normal endometrium. Data from sheep shows that ASCT2 expression was induced in the endometrium in response to interferon tau and prostaglandins, indicating a role for ASCT2 in peri-implantation pregnancy.^42, 43^ Here we have clearly implicated ASCT2 in the regulation of proliferation in Ishikawa and HEC1A cells. It is possible that ASCT2 upregulation could represent a normal regulatory mechanism for endometrial cell proliferation under certain conditions such as pregnancy. Indeed, in prostate cancer, ASCT2 can be regulated by androgen receptor signalling, and it is therefore possible that pregnancy hormones may also transcriptionally regulate ASCT2 expression. Moreover this mechanism could be co-opted by cancer cells allowing cancer cell growth.

We have shown that ASCT2 is expressed in all four endometrial cancer cell lines tested. The highest expression level was seen in HEC1A cells and the lowest in KLE cells. There appeared to be some association between ASCT2 expression, glutamine uptake and cell proliferation, with the exception of Ishikawa cells. KLE showed the lowest expression of ASCT2 at the mRNA and protein level. Conversely KLE showed upregulation of glutamine metabolism enzymes GLS and CTPS and trended towards upregulation of GMPS also (Figure 3f; Supplementary Figure 2B). This upregulation, together with expression of SNAT1 and SNAT2, may help to compensate for the reduced expression of ASCT2 in KLE cells. It may also indicate that despite low ASCT2 expression, KLE is more highly dependent on glutamine metabolism than other endometrial cancer cell lines. The expression of an alternative glutamine transporter, SNAT1, was significantly lower in HEC1A compared to Ishikawa and a similar trend was observed with SNAT2 expression (*P*=0.0597). HEC1A also shows significantly higher expression of CTPS1, CTPS2 and GMPS than Ishikawa cells (Figure 3f; Supplementary Figure 2B). This may indicate that HEC1A cells are more dependent on ASCT2-mediated glutamine uptake for pyrimidine and purine biosynthesis, than Ishikawa cells. This could contribute to the differences seen between the effects of ASCT2 knockdown on spheroid area in these two cell lines (Figures 6d and h). Conversely, ASCT2 knockdown resulted in reduced proliferation of both cell lines indicating ASCT2-mediated glutamine transport contributes to cellular proliferation in both cell types.

Chemical inhibition of ASCT2 inhibits growth of endometrial cancer cell lines *in vitro*. We have used two different chemical inhibitors, BenSer and GPNA. GPNA was effective in Ishikawa, HEC1A and KLE, while BenSer was able to inhibit cell growth to a greater extent than GPNA in Ishikawa, HEC1A and RL95-2, despite causing a similar reduction in glutamine uptake. Previously we have shown that BenSer is a more promiscuous amino acid transporter inhibitor than GPNA, and is also capable of inhibiting leucine transport via LAT1/SLC7A5.^22^ This is consistent with our finding that LAT1 also plays a role in endometrial cancer cell growth,^39^ and explains the larger effects of BenSer on cell proliferation. Recently, GPNA was also shown to also block the glutamine transporters, SNAT1 and SNAT2.^31^ Furthermore, CRISPR knockout of ASCT2 in the osteosarcoma cell line 143B resulted in a compensatory upregulation of the SNATs, which may explain the smaller effects seen with stable ASCT2 knockdown compared to GPNA or BenSer inhibition.^31^ Consistent with a role for other glutamine transporters in endometrial carcinoma we found that SNAT1 is upregulated in endometrial carcinoma compared to normal aged matched endometrium (Supplementary Figure 1A). In addition, both SNAT1 and SNAT2 were significantly elevated in serous carcinoma compared to endometrioid adenocarcinoma of the uterus (Supplementary Figure 1B and D). This indicates that a broader inhibition of the glutamine uptake mechanisms may be a better strategy for targeting this glutamine-dependent growth-promoting pathway in endometrial cancer cells. Importantly, we have shown using ASCT2 shRNA that ASCT2 directly contributes to cell proliferation in Ishikawa and HEC1A cells and spheroid size in HEC1A. This confirms a role of ASCT2 in the regulation of endometrial cancer cell proliferation in some cell lines. The additional effects of BenSer and GPNA compared to ASCT2 knockdown also suggest complementary roles for other glutamine transporters SNAT1 and SNAT2 in endometrial carcinoma.

Neither BenSer or GPNA consistently resulted in changes in apoptosis, indicating that ASCT2 is important for endometrial cancer cell growth but not necessarily survival. This is in contrast to data seen in 3D culture, where GPNA was more effective than BenSer at reducing endometrial spheroid cross-sectional area. In addition, GPNA appears to reduce cell viability in 3D culture, due to the presence of compacted nuclei, typical of apoptosis, in GPNA treated spheroid cultures. Peripheral cell staining of ASCT2 was more evident when cells were cultured in conditions producing 3D spheroids. This was focussed to the apical surface of the spheroids, which is protected from the environment by the formation of tight junctions.^32^ The induction of cell death by GPNA and not BenSer could therefore be due to differences in the ability of drugs to penetrate 3D spheroids. These data indicate that cellular context alters the subcellular localisation of ASCT2 and the response to chemical inhibition. This could be an important consideration when studying the role of amino acid transporters in tumours derived from glandular epithelium *in vitro*. It is important to note that these data may be confounded by the conditions of the 3D culture system, which was serum-free and contained only a defined set of nutrients and growth factors.^32^ Therefore GPNA may be able to reduce cell viability under certain culture conditions, but it is not clear whether this would be relevant to endometrial cancers *in vivo*. This is supported by recent data in prostate and triple-negative breast cancer cells, where *in vitro* ASCT2 knockdown has lower effects on cell growth, compared to *in vivo* knockdown. This most likely relates to the abundance of amino acids in tissue culture media compared to poorly vascularised tumours *in vivo.*^20, 21^

In this study we have shown that ASCT2 function contributes to cancer cell proliferation in a subset of endometrial cancer cell lines. Because ASCT2 expression contributes to cell growth, this raises the possibility that drugs targeting ASCT2, or more broadly cellular glutamine uptake or downstream glutamine enzymes, could be utilised as a therapeutic strategy for the treatment of endometrial carcinoma. In triple-negative breast cancer, the glutaminase inhibitor CB-839 is effective both as a single agent and in combination with paclitaxel,^44^ and has entered clinical trials. Recently, the CAD inhibitor leflunomide was shown to combine with doxorubicin in mouse models of triple-negative breast cancer.^45^ Additionally, the GMPS inhibitor decoyinine showed anti-tumour efficacy in melanoma xenografts.^46^ Low grade endometrial carcinoma are typically treated with surgery involving hysterectomy which results in a loss of fertility; and salpingo-oophorectomy which leads to long-term sequelae associated with oestrogen deprivation.^2, 47^ Higher-grade tumours, which include serous carcinomas, often also require additional radiotherapy and/or chemotherapy.^3^ Therefore there is a need for more targeted therapies against endometrial carcinoma to treat more aggressive disease and to improve outcomes for younger patients who face long-term consequences of currently available therapies.

## Materials and methods

### Gene expression analysis

Human primary endometrial cancer gene expression data was downloaded from GEO (http://www.ncbi.nlm.nih.gov/geo/; GDS4589)^29^ and cBioPortal http://www.cbioportal.org/).^30^

### Immunohistochemistry

Primary endometrial carcinoma samples were obtained from Royal Prince Alfred Hospital, Australia, in accordance with ethics approval from the Sydney Local Health District Ethics Board (X13-0195). All tumours were classified according to current FIGO classifications.^48^ Sections from formalin-fixed paraffin-embedded tissue were stained using an automated immunohistochemistry staining processor (VENTANA BenchMark ULTRA, Tucson, AZ, USA). CC1 epitope retrieval buffer was used for 64 min before application of the primary antibody ASCT2 (polyclonal, 1:200, Transgenic, Kobe, Japan) for 1 h. Each sample was scored by staining intensity as weak to strong in normal and neoplastic cells by a clinical pathologist (L.A.). The distribution of staining was recorded as negative (<5% of cells stained), 1+ (5–25%), 2+ (26–50%) or 3+ (>50%) based in part on Allred semi-quantitative scores of nuclear protein expression used elsewhere.^49, 50, 51^

### Cell culture

The human endometrial cancer cell lines used were Ishikawa, RL95-2, HEC1A, and KLE (kind gift from Associate Professor Deborah Marsh of the Kolling Institute, Sydney, NSW, Australia). Cell identity was confirmed by short tandem repeat profiling in 2014 (CellBank, Westmead, NSW, Australia). Cells were maintained at low passage from these authenticated stocks, and routinely tested as free from mycoplasma contamination. Cells were cultured in DMEM:F12 media (Life Technologies, Carlsbad, CA, USA) containing 10% (v/v) FBS (Scientifix, Cheltenham, VIC, Australia) and penicillin-streptomycin solution (Sigma, St Louis, MO, USA), at 37 °C and 5% CO_2_. Spheroid culture was performed according to Eritja *et al.*^32^ Briefly, 8 chamber Lab-Tek II chambered #1.5 German coverglass (Nunc, Roskilde, Denmark) were coated in Matrigel (Corning, Corning, NY, USA). Cells (2 × 10^4^) were plated in DMEM:F12 (Life Technologies) containing penicillin-streptomycin, 1 mM HEPES, 5 ng/ml EGF, 1:100 dilution of Insulin-Transferrin-Selenium (ITS) supplement (Life Technologies) and 3% Matrigel for 8 days with 3-4 media changes. ASCT2 shRNA knockdown was performed as previously described.^22^ Briefly, endometrial cancer cell lines were transduced with lentiviral constructs containing shControl (ath-miR159a) or shASCT2 (two sequences; sh 28 or sh 63^22^) for 2 days followed by puromycin (2 μg/ml) selection. Cultures were subsequently maintained in 2 μg/ml puromycin to maintain shRNA expression.

### Antibodies for western blotting and immunofluorescence

Antibodies used for western blotting and immunofluorescence were rabbit anti-ASCT2 (Cell Signaling, Danvers, MA, USA, 1:100 for immunofluorescence and 1:1000 for western blot), and mouse anti-GAPDH (ab8245, Abcam, 1:5000 for western blot), immunofluorescent images were counterstained with 1:200 rhodamine-phalloidin (Life Technologies) and/or 200 ng/ml DAPI. Western blots were performed in three separate experiments and representative results shown. Densitometric analysis was performed using Fiji (ImageJ; NIH, Bethesda, MA, USA) software. Immunostaining was performed on duplicate wells in three separate experiments, 7–13 spheroids were imaged per well. Condensation of nuclei as detected by DAPI in a manner characteristic of apoptosis was used as an indicator of cell viability. Images were acquired using a Deltavision Elite Microscope (GE Healthcare, Little Chalfont, UK) and deconvolved using Softworx Software (GE Healthcare). Cross-sectional area was determined using Volocity Software (PerkinElmer, Waltham, MA, USA).

### Quantitative RT–PCR

Quantitative reverse transcriptase PCR (RT–qPCR) was performed using a BioRad CFX96 Real-Time System (BioRad, Hercules, CA, USA) using SYBR green detection with the following primer pairs: ASCT2: 5′-GTGGCGCTGCGGAAGCT-3′ and 5′-GGCGTACCACATGATCCAG-3′, SNAT1: 5′-AATGCAATGGTGGGTAAAGC-3′ and 5′-TGACACGTGTACGCCAAAAT-3′, SNAT2: 5′-GCTGCAGATGCACCAATAAA and 5′-TTACTTGTCATCTTTGTCCCAAC-3′, GLS: 5′-CAATTGCTGAAGGACAAGAGAA-3′ and 5′-CAGACGTTCGCAATCCTGTA-3′, CTPS1: 5′-ACTTGGGGAAAACTGTCCAA-3′ and 5′-CTCATCACCCACTCCTGGAT-3′, CTPS2: 5′-TTCTCCACCATCATTTAAGACG-3′ and 5′-ATCGATGCTGGCACTTTTTC-3′, CAD: 5′-AGTCTCGGCACAGCTGACTT-3′ and 5′-CTCAAAGTGCCAGTCCACAG-3′, PFAS: 5′-CACTGTGGAGGCCTTTGACT-3′ and 5′-GCCCTTGAAGAACCAGTGTC-3′, GMPS: 5′-TGGCACGTTCTGGAAACATA-3′ and 5′-ACTTCAGGGTGGAACTGTGC-3′, PPAT: 5′-TGGTGTGTCCAATTCCAAGA-3′ and 5′-TCACACAAGGGAATGGGTCT-3′. Relative gene expression was calculated using ΔΔCT normalised to GAPDH gene expression using the following primers: 5′-TGCACCACCAACTGCTTAGC-3′ and 5′-GGCATGGACTGTGGTCATGAG-3′.

### Glutamine uptake assay

[^3^H]-l-glutamine uptake was conducted as previously described.^22^ Briefly, cells (3 × 10^5^) were cultured in DMEM:F12 media and incubated in the absence or presence of 10 mm BenSer (BenzylSerine) or 1 mm GPNA (gamma-l-Glutamyl-P-NitroAnilide) for 30 min at 37 °C, before addition of [^3^H]-l-glutamine (200 nm; PerkinElmer) in glutamine-free MEM media for a further 30 min. For shRNA uptake experiments, cells were incubated with [^3^H]-l-glutamine (400 nm; PerkinElmer) for 15 min. Cells were collected, transferred to filter paper using a 96-well plate harvester (Wallac PerkinElmer), dried, exposed to scintillation fluid and counts measured using a liquid scintillation counter (PerkinElmer).

### Cell viability assays

MTT (Millipore, Billerica, MA, USA) assays were conducted as per the manufacturer’s instructions and described previously^52^ using 100–250 cells per well in a 96-well plate. Fresh media with vehicle, 10 mm BenSer or 1 mm GPNA were applied every two days.

### Flow cytometry

Cells (1–2 × 10^5^) were cultured for 24 h followed by treatment where stated with vehicle, 10 mm BenSer or 1 mm GPNA for 24 h. Staining for flow cytometric analysis was performed as previously described.^22^

### Statistical analysis

The data are expressed as mean±s.e.m. The two-tailed student’s *t*-test was used to compare differences among groups. The Mann–Whitney *U*-test was used for non-parametric gene expression data. Outlier analysis of the Risinger *et al.*^53^ cohort was performed using the ROUT function in Prism 6 (GraphPad, La Jolla, CA, USA).
