# Supplementary material for: ASCT2 regulates glutamine uptake and cell growth in endometrial carcinoma
Source: Oncogenesis. 2017 Jul 31;6(7):e367–. doi: 10.1038/oncsis.2017.70 (PMC5541720; doi:10.1038/oncsis.2017.70)
Supplement: Supplementary Figure 1 [file oncsis201770x1.pdf]

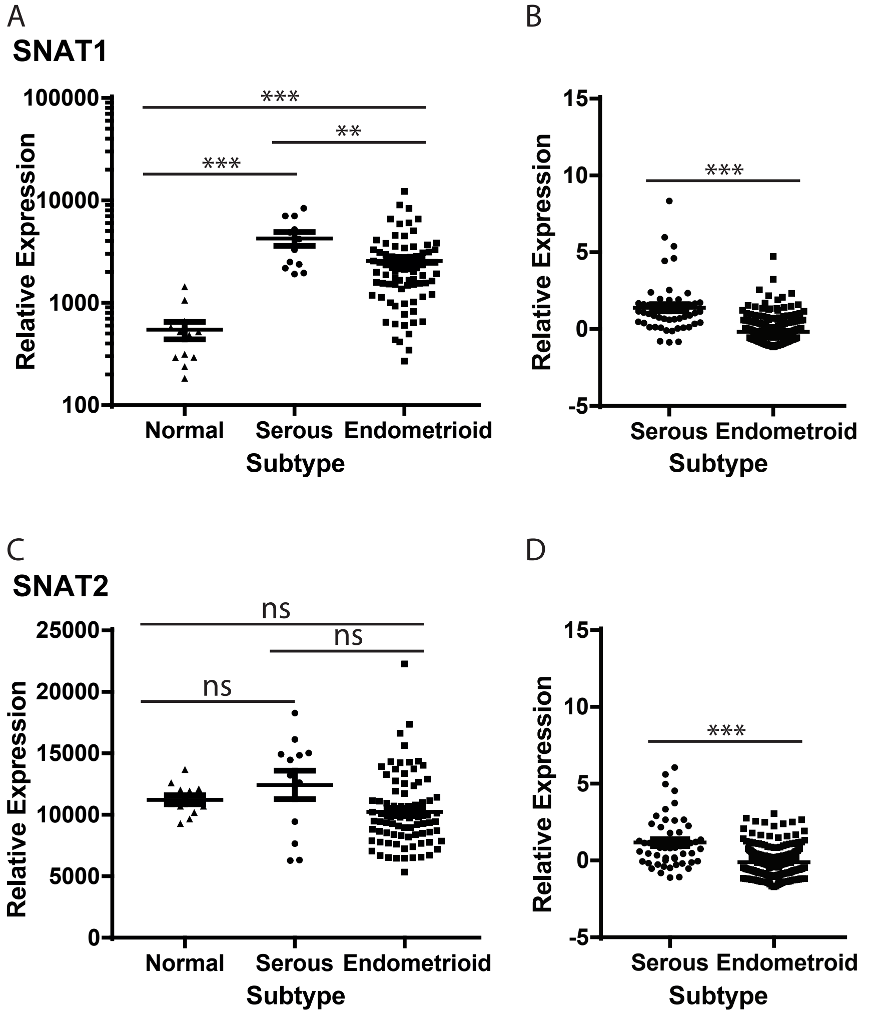

Supplementary Figure 1: SNAT1 and SNAT2 expression in serous and endometrioid endometrial carcinoma subtypes

SNAT1 (A) and SNAT2 (C) expression in serous and endometrioid tumours compared to normal endometrium from age matched controls derived from the Risinger et al gene expression cohort.(29) Normal n=12; Serous n=12, Stage I, Grade 3; Endometrioid n=79, Stage I, Grade 1-3. Mann-Whitney U-test: *** P<0.001, ns P>0.05. SNAT1 (B) and SNAT2 (D) expression in serous and endometrioid subtype tumours in the TCGA invasive endometrial carcinoma cohort.(30) Serous n=52, Stage I-IV, Grade 3; endometrioid n=271, Stage I-IV, Grade 1-3; Mann Whitney U-test: ns P>0.05.
