# Supplementary material for: ASCT2 regulates glutamine uptake and cell growth in endometrial carcinoma
Source: Oncogenesis. 2017 Jul 31;6(7):e367–. doi: 10.1038/oncsis.2017.70 (PMC5541720; doi:10.1038/oncsis.2017.70)
Supplement: Supplementary Figure 2 [file oncsis201770x2.pdf]

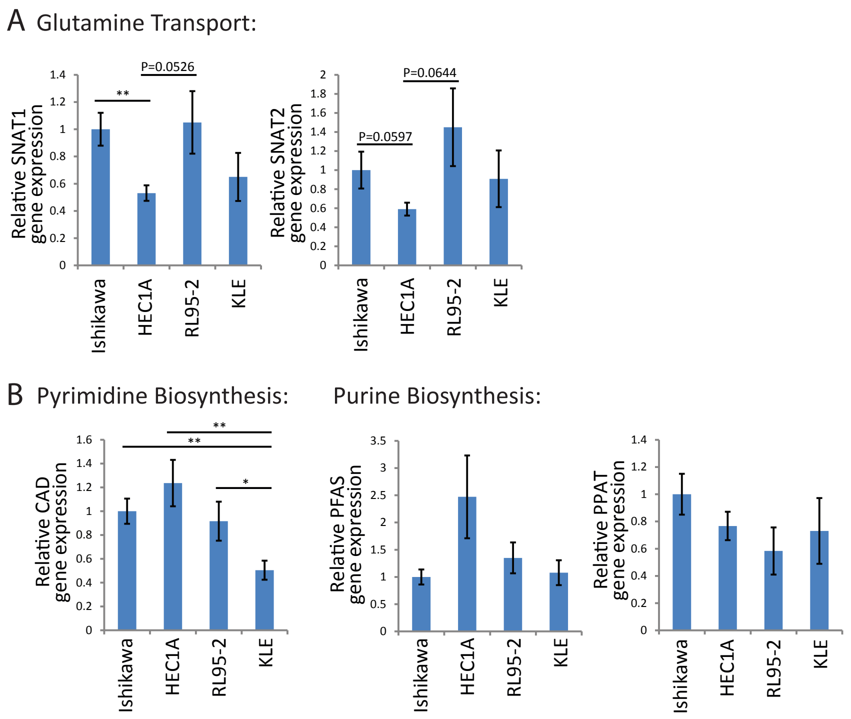

Supplementary Figure 2: Expression of glutamine transporters and downstream enzymes in endometrial cancer cell lines.

RT-qPCR data showing the relative mRNA expression of glutamine transporters SNAT1 and SNAT2 (A), pyrimidine biosynthesis enzyme CAD (B) and purine biosynthesis enzymes PFAS and PPAT (B) in endometrial cancer cell lines, n=5-6. * P<0.05, ** P<0.01 and *** P<0.001 Student's t-test.
