# Supplementary material for: ASCT2 regulates glutamine uptake and cell growth in endometrial carcinoma
Source: Oncogenesis. 2017 Jul 31;6(7):e367–. doi: 10.1038/oncsis.2017.70 (PMC5541720; doi:10.1038/oncsis.2017.70)
Supplement: Supplementary Table [file oncsis201770x3.docx]

**Supplementary Table 1. Primary human endometrial cancer cohort stained for ASCT2.**

Primary human endometrial cancer samples were stained with an ASCT2 antibody. The staining intensity was graded as weak or strong in normal and neoplastic cells. The distribution of staining was recorded as negative (<5% of cells stained), 1+ (5%-25%), 2+ (26%-50%) or 3+ (>50%).

| Sample | Tumour subtype | FIGO Grade | Observations |
| --- | --- | --- | --- |
| 001 | endometrioid | 1 | Varies from 0 to 3+ cytoplasmic staining |
| 002 | endometrioid | 2 | Really unusual staining pattern, surface cancer cells positive 3+ cytoplasmic, basal cancer cells negative |
| 003 | endometrioid | 1 | Discreet staining of neoplastic endometrium 3+, whole cytoplasm, non-granular, normal glands negative |
| 004 | endometrioid | 3 | 3+ cytoplasmic staining throughout |
| 005 | endometrioid | 1 | Dichotomous staining pattern, normal negative, most abnormal 3+ cytoplasmic but some negative |
| 006 | endometrioid | 1 | 3+ cytoplasmic staining in tumour, negative in normal glands, positive in endometrial stromal cells of adenomyosis |
| 007 | endometrioid | 1 | 3+ cytoplasmic staining in tumour, negative in most normal glands, negative in some that look like CAH |
| 008 | endometrioid | 3 | Dichotomous staining pattern, most abnormal 3+ cytoplasmic but some solid areas of tumour are negative |
| 009 | endometrioid | 3 | 3+ cytoplasmic staining in tumour, negative in normal glands, positive in endometrial stromal cells |
| 010 | endometrioid | 3 | 3+ cytoplasmic staining in tumour, no non-neoplastic glands for comparison |
| 011 | endometrioid | 3 | 3+ cytoplasmic staining in 90% of tumour, some negative areas |
| 012 | endometrioid | 3 | Normal glands negative, endometrial stromal cells positive, cancer has 2-3+ cytoplasmic staining but some negative too |
| 013 | endometrioid | 2 | Mostly 3+ cytoplasmic staining in tumour, occasional areas of loss per some glands |
| 014 | endometrioid | 2 | 3+ cytoplasmic staining in tumour |
| 015 | endometrioid | 2 | 3+ cytoplasmic staining in tumour |
| 016 | endometrioid | 1 | 3+ cytoplasmic staining in tumour |
| 017 | endometrioid | 1 | Very heterogeneous staining pattern from tumour from 3+ to patchy cells only; possible staining in CAH |
| 018 | endometrioid | 3 | Very variable 3+ to patchy across tumour |
| 019 | endometrioid | 3 | Mostly 3+ cytoplasmic in tumour, normal glands are negative, some areas of more patchy staining |
| 020 | endometrioid | 2 | Mostly 3+ cytoplasmic in tumour, focal areas show increased basal staining |
